# A case of adenocarcinoma of the rete testis with durable response to cisplatin‐based chemotherapy

**DOI:** 10.1002/iju5.12298

**Published:** 2021-06-19

**Authors:** Shunsuke Owa, Takeshi Sasaki, Katsunori Uchida, Susumu Watanabe, Momoko Kato, Yusuke Sugino, Manabu Kato, Satoru Masui, Kouhei Nishikawa, Yuko Yoshio, Hideki Kanda, Yoshiki Sugimura, Takahiro Inoue

**Affiliations:** ^1^ Department of Nephro‐Urologic Surgery and Andrology Mie University Hospital Tsu Mie Japan; ^2^ Department of Pathology Mie University Hospital Tsu Mie Japan

**Keywords:** adenocarcinoma of the rete testis, cisplatin‐based chemotherapy

## Abstract

**Introduction:**

Adenocarcinoma of the rete testis is a rare malignancy with a poor prognosis. We report a case of adenocarcinoma of the rete testis with a durable response to cisplatin‐based chemotherapy.

**Case presentation:**

A 48‐year‐old man with Down syndrome (trisomy 21) presented with a 1‐month history of painless swelling of the left scrotum. The physical examination revealed a left testis with a hydrocele associated with a tumor and enlarged pelvic and para‐aortic lymph nodes. He underwent a radical orchiectomy. The specimen was diagnosed as adenocarcinoma of the rete testis. The patient received 7 cycles of chemotherapy (1 cycle of BEP and 6 cycles of EP) postoperatively. The metastatic lymph nodes were reduced in size for at least 12 months. Our patient with adenocarcinoma of the rete testis obtained an acceptable response to cisplatin‐based chemotherapy.

**Conclusion:**

We treated a patient with an adenocarcinoma of the rete testis who had an acceptable response to platinum‐based chemotherapy.

**Abbreviations & Acronyms**
AFP: α‐fetoprotein
AORT: adenocarcinoma of the rete testis
BEP: bleomycin, etoposide, and cisplatin
CEA: carcinoembryonic antigen
CT: computed tomography
EP: etoposide and cisplatin
hCG: human chorionic gonadotropin
RPLND: retroperitoneal lymph node dissection

**Keynote message**

AORT has a poor prognosis. There is no established cure. We treated a patient with AORT who obtained a durable response to cisplatin‐based chemotherapy.

## Case report

**Fig. 1:**
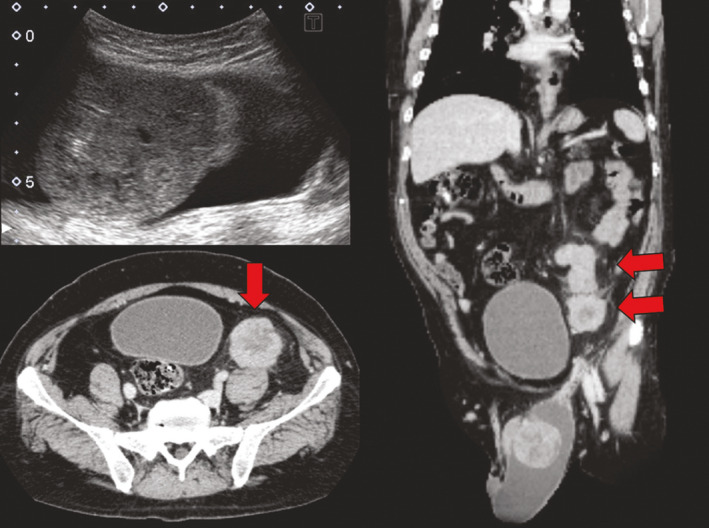
Ultrasonography showed testicular enlargement with a mosaic appearance inside the hydrocele of the testis, which was suspicious for a testicular tumor. CT showed a testicular tumor with a maximum diameter of 6.2 cm inside the hydrocele of the testis. Metastases were found in the pelvic and pararenal aorta lymph nodes, with a maximum diameter of 6.1 cm.

The patient was a 48‐year‐old man with Down syndrome (trisomy 21) who was evaluated in our clinic with a 1‐month history of painless swelling involving the left scrotum. He had a history of hyperthyroidism and hyperuricemia. Ultrasonography revealed a left testicular mass and hydrocele. CT showed a 5.6 × 6.0 × 6.2 cm left testicular tumor within the hydrocele and a total of 8 enlarged lymph nodes, the largest of which was 6.1 cm in the pelvis and pararenal aorta (Fig. 1). The lactate dehydrogenase, AFP, total serum hCG, and CEA levels were within the normal range.

**Fig. 2:**
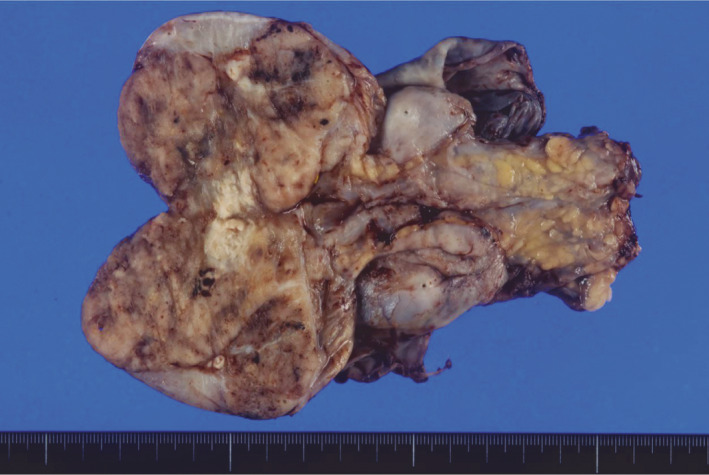
Macroscopic findings. A dark red tumor showing small hemorrhagic areas was observed within the resected specimen. The tumor measured 45 × 50 × 60 mm.

**Fig. 3:**
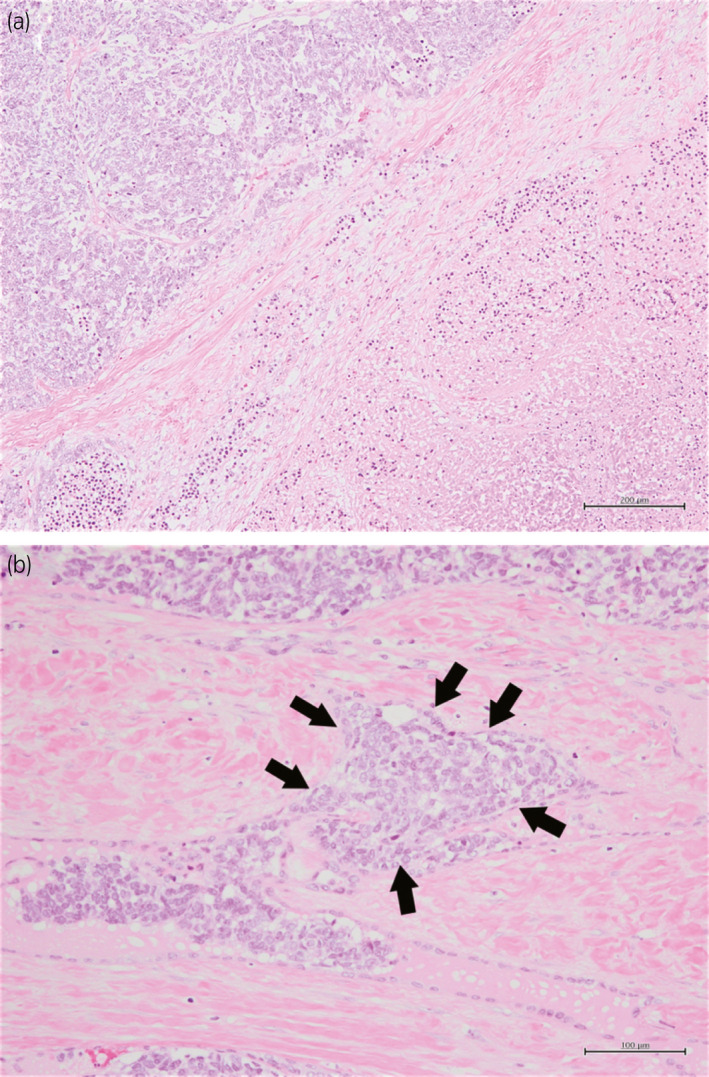
(a) Histologically, tumor cells with a high nuclear‐to‐cytoplasm ratio grew in a sheet‐like pattern and showed extensive necrosis (bar = 200 µm). (b) Small foci of proliferating spindle‐shaped tumor cells are seen. *In situ* lesions were recognized in the rete testis (arrows) (bar = 100 µm).

The patient underwent a radical orchiectomy. The specimen measured 45 × 50 × 60 mm (Fig. 2). Grossly, the tumor was located in the testis and para‐testis. There was no apparent involvement of the surface of tunica vaginalis. Histopathologicaly, the tumor was located in the testis and rete testis. High‐grade tumor cells with a high nuclear‐to‐cytoplasm ratio and prominent nucleoli showed solid, nested, and sheet‐like growth patterns with extensive areas of necrosis (Fig. 3a). Small foci of spindle‐shaped tumor cells were observed. The spindle cell nuclear chromatin was finely granular and nucleoli were absent. Carcinoma *in situ* was observed in the rete testis (Fig. 3b). There was no germ cell tumor *in situ* in the testis. Immunohistochemical staining was positive for AE1/AE3, calretinin (focal), PAX8 (focal), and CAⅨ expression. Immunohistochemical staining was negative for WT1, AFP, OCT3/4, PLAP, myogenin, s100a, STAT6, EMA, CK7, CK20, GATA3, CD34, CD56, CD99, SALL4, HMB45, chromogranin A, and synaptophysin expression. The results of hematoxylin‐eosin staining and immunostaining ruled out germ cell, sex cord, and stromal tumors. The absence of *in situ* mesothelial involvement and WT1 negativity ruled out mesothelial and serous neoplasms. The absence of tumor invasion and *in situ* lesions in the epididymis ruled out the tumors of epididymal origin. The diagnosis of AORT was based on the presence of *in situ* lesions in the rete testis and the exclusion of other diseases considered in the differential diagnosis.

The postoperative diagnosis of the findings was AORT with pelvic and para‐aortic lymph node metastases (a total of 8 pieces with a maximum diameter of 6.1 cm). The clinical stage according to the testicular tumor was cT3N1M0S0, stage IIA. We planned to administer four courses of BEP based on our treatment of testicular tumors^1^; however, he developed severe side effects during the first course of BEP. After a discussion with the oncologist, it was decided to switch to a reduced dose of EP and continue treatment.

**Fig. 4:**
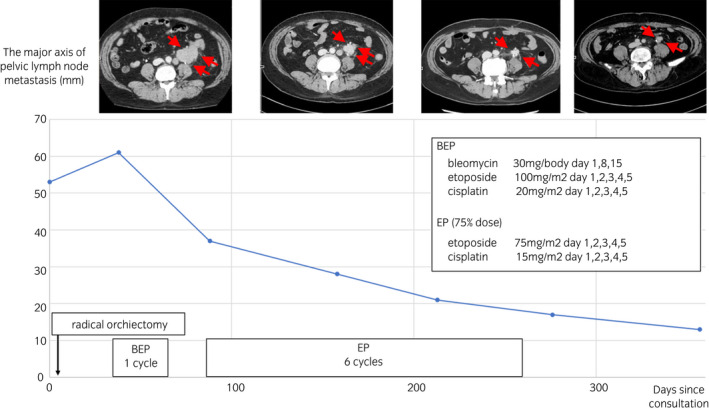
Clinical course of the case. Chemotherapy was administered after orchiectomy. After the end of chemotherapy, the lymph node metastases continued to shrink.

The lymph node metastases shrank after the first chemotherapy cycle. After the sixth course of chemotherapy, the patient’s family wished to discontinue the treatment because of his physical weakness, and he was monitored by CT every 3 months thereafter. The family did not wish to have the residual tumor removed. The patient has remained free of lesions, and his metastatic lymph nodes have not enlarged up to 12 months after the orchiectomy and 7 months after the chemotherapy was discontinued (Fig. 4). CT follow‐up was also terminated due to the patient’s unstable mental state. The patient is alive 20 months after discontinuing chemotherapy.

## Discussion

AORT is a rare tumor with a poor prognosis. The 3‐ and 5‐year disease‐free survival rates of AORT are 49% and 13%,^2^ respectively. The actual incidence of AORT is unknown. Thirty‐two cases of AORT have been reported in the English literature since 2000. Sánchez‐Chapado *et al*.^2^ reported that AORTs occur more frequently on the right side (1.5:1); however, of the 32 reported cases, 15 occurred on the right side and 15 on the left side with no apparent predilection for the right and left sides. Two cases were unknown.

Studies using animal models have shown an association between AORT and chemicals, such as diethylstilbestrol^3^; however, such an association has not been demonstrated in humans. There have been reports of AORT secondary to adenomatous hyperplasia. Whether the association between adenomatous hyperplasia and AORT is important has not been established.^4^

The most common clinical manifestation of AORT is scrotal swelling (94%), with 27% of patients having associated scrotal pain and 19% presenting with signs and symptoms related to metastases.^5^ Hydrocele and chronic epididymitis are often associated with this tumor. Among the 32 reported cases, hydroceles co‐existed in 14 (43%). The high prevalence of a concomitant hydrocele might be accounted for by the location of the tumor, which could result in lymphatic perfusion and blood flow obstruction in the testes.^6^

Considering the differential diagnosis and distinguishing between AORT and germ cell tumors of the testis, most previous reports of AORT did not show elevated serum AFP or hCG levels, thus suggesting germ cell tumors (especially non‐seminomas). Indeed, some reports on AORT have revealed serum CEA as an alternative marker.^7^ The tumor marker levels were not elevated in our patient. Thus, differentiating between AORT and germ cell tumors is difficult without performing a histopathologic evaluation.

AORT exhibits various histologic patterns. The majority of cases exhibit a mixed growth pattern. Table 1 shows the histopathologic characteristics in 25 of 32 cases in which histologic findings were reported. The most common documented pattern is papillary, followed by tubulopapillary, glandular, and solid. Slit‐like, nested, glomeruloid, micropapillary, cribriform, and biphasic patterns have been reported less frequently.^8^ Amin^9^ described the diagnostic features of AORT based on the report by Nochomovitz and Orenstein,^10^ as follows: (i) neoplasm centered in the hilum of the testis; (ii) microscopic morphology incompatible with other testicular/paratesticular neoplasms; (iii) absence of histologically similar extra‐scrotal primary neoplasms; and (iv) immunohistochemical exclusion of other possibilities (most notably malignant mesothelioma and papillary serous carcinoma).

**Table 1:** Histopathological characteristics of AORT

Characteristics	Cases	*n* (%)	Our case
A mixed growth pattern	20	80	〇
A single growth pattern	5	20	
Papillary	23	92	
Glandular	15	60	
Solid	12	48	〇
Tubular	9	36	
Trabecular	5	20	
Glomeruloid	5	20	
Nested	4	16	〇
Cribriform	4	16	
Sheet	3	12	〇
Micropapillary	2	8	
Necrosis	9	36	

Because AORTs are rare, the specific immunohistochemical characteristics have not been established. Previous reports have shown that the expression of AE1/AE3, Ber‐EP4, EMA, CK7, vimentin, and MOC31 is often positive. The expression of PAX8, CK5/6, WT1, calretinin, and CK20 can frequently be positive.^8^ In our case, we were able to exclude mesothelioma and other testicular and metastatic tumors from the diagnosis based on the results of immunohistochemistry and lack of tumor *in situ* in the serosal membrane. Although the most common growth pattern, a papillary structure, was not observed, our case showed a mixed growth pattern, with solid, nested, and sheet‐like patterns. In addition, carcinoma *in situ* in the rete testis was demonstrated. The diagnosis of AORT was made because the histopathologic findings were consistent with previously reported findings and met the diagnostic criteria reported by Nochomovitz *et al*.^10^

AORT had been previously reported to have a poor prognosis. There is no standard chemotherapy for AORT.^8^ Among the 32 cases reported since 2000, chemotherapy was administered to 9. Table 2 summarizes the reported chemotherapy regimens and the outcomes. Our patient survived for a long time after discontinuing chemotherapy. He survived for 20 months even though chemotherapy was discontinued and no residual tumor was resected, suggesting that there were no viable cells remaining. Based on our experience, we suggest that platinum‐based chemotherapy may be effective for AORTs. A BEP regimen was considered as chemotherapy for AORT in accordance with the recommended treatment for other testicular tumors. Not only does BEP contain platinum, but urologists may find it easier to use because of their experience with BEP.

**Table 2:** Summary of chemotherapy regimens previously administered in the literature

	Lymph node metastasis	Distant metastasis	Time‐tometastasis	Chemotherapy	Regimen	Cycles	Follow‐up period (m)	Outcome
Musser *et al*.^11^	Yes	No	7 months after surgery	After recurrence	Details unknown	Unknown	36	Alive
Lee *et al*.^12^	Yes	No	At diagnosis	Salvage	Details unknown	6	8	Died of disease
Rubegni *et al*.^13^	Yes	No	At diagnosis	Salvage	BEP	4	12	Alive
Lin *et al*.^14^	No	No	At diagnosis	Adjuvant	BEP	2	15	Alive
Spataro *et al*.^15^	No	No	At diagnosis	Adjuvant	Platinum base	4	12	Alive
Tohru *et al*.^16^	Yes	Yes	At diagnosis	8 months before surgery	Paclitaxel and carboplatin	2	8	Died of disease
	No	Yes	At diagnosis	Salvage	Details unknown	Unknown	12	Died of disease
Khaller *et al*.^2^	Yes	No	At diagnosis	Salvage	Details unknown	Unknown	8	Alive
	Yes	No	At diagnosis	Salvage	Details unknown	Unknown	Unknown	Unknown
Our case	Yes	No	At diagnosis	Salvage	BEP	1	20	Alive
					EP	6		

Surgical excision of the tumor is the most effective treatment. Metastases to the lymph nodes often occur, and metastases to the lungs, liver, and bone have also been frequently reported.^8^ According to the report by Sanchez‐Chapado *et al*.^2^, the overall survival of patients with tumor diameters <5 cm is better than the overall survival of patients with larger tumors. Performing an orchiectomy in near future is important.

Whether RPLND changes the outcome is unclear because the tumor is too rare for prospective studies. Based on a retrospective literature review of published cases, Sanchez‐Chapado *et al*.^2^ concluded, however, that prophylactic RPLND appeared to provide a survival advantage. Given the poor response of the tumor to chemotherapy, the addition of RPLND to orchiectomy might provide not only useful information on the extent of disease, but also the possibility that micrometastases are resected. Of the 32 cases reported since 2000, 6 patients underwent RPLND and 5 were still alive at the time the report was published. Among the patients who underwent RPLND, however, only 1 had positive lymph nodes among the resected specimens, despite a negative PET‐CT scan.^17^ Additional cases are required to determine if RPLND is beneficial.

We have shown that platinum‐containing chemotherapy may be effective in the postoperative treatment of patients with an AORT. Furthermore, we consider it very rare that a response was obtained despite limited chemotherapy. The order in which chemotherapy and surgical treatment, such as RPLND, should be administered is an issue that needs to be addressed in future.

## Conclusion

We treated a patient with an AORT who had an acceptable response to platinum‐based chemotherapy. Additional cases are needed to provide information on the optimal chemotherapy regimen.

## Conflict of interest

The authors declare no conflict of interest.
